# Effect of high phytase levels on growth performance and calcium and phosphorus metabolism in broilers fed a non-inorganic phosphorus diet

**DOI:** 10.3389/fphys.2026.1944916

**Published:** 2026-09-09

**Authors:** Yanling Chen, Ying Gao, Xiaojuan Wang, Hongchao Jiao, Jingpeng Zhao, Haifang Li, Yunlei Zhou, Minghui Wang, Min Liu, Kelin Li, Hai Lin

**Affiliations:** 1 Department of Animal Science, Shandong Agricultural University, Taian, Shandong, China; 2 College of Life Sciences, Shandong Agricultural University, Taian, Shandong, China; 3 College of Chemistry and Material Science, Shandong Agricultural University, Taian, Shandong, China

**Keywords:** bone development, broiler, calcium and phosphorus metabolism, growth performance, inorganic phosphorus, phytase

## Abstract

The effects of high phytase levels on growth performance and calcium (Ca) and phosphorus (P) metabolism in broilers fed non-inorganic P diets were investigated. A total of 600 one-day-old broilers were assigned to either a control diet or a non-inorganic P diet supplemented with one of two commercial phytases (P1 and P2) at 1,000 (NP, normal level of phytase) or 10,000 FTU/kg (HP, high level of phytase). Compared with the control group, HP treatments had comparable feed intake, body weight gain and feed conversion ratio, whereas NP supplementation reduced the parameters, particularly during 22–42 and 1–42 days of age (*p* < 0.05). Both HP and NP treatments significantly increased apparent P retention during the finisher stage, compared with the control group (*p* < 0.01). At 42 days of age, compared with the control group, the HP chickens had higher tibial and femoral Ca contents (*p* < 0.01) and comparable P levels, whereas the NP chickens showed lower Ca and P contents (*p* < 0.01). High phytase levels increased blood concentrations of P and PTH, along with tibial and femoral lengths. Compared with the control group, phytase altered the expression of genes involved in Ca and P transport. NP treatment increased duodenal *CaBP-D28k* expression, while the P1 treatment at 1,000 FTU/kg significantly upregulated renal *NPt2a* and *CaBP-D28k* expression (*p* < 0.01). In conclusion, supplementation with 10,000 FTU/kg phytase to corn–soybean meal diet without inorganic P supplementation largely maintained growth performance and bone mineralization while improving P utilization and reducing P excretion. These findings indicate that 10,000 FTU/kg phytase can effectively replace inorganic P supplementation in broilers.

## 1 Introduction

Phosphorus (P) is a finite resource, raising concerns about long-term global P supply security (Walsh et al., 2023). As an essential mineral, P plays a critical role in bone mineralization, enzyme activation, energy metabolism, and cellular signaling (Omotoso et al., 2023). In plant ingredients, P is primarily bound to phytate, which limits its utilization by monogastric animals (Selle et al., 2023). Meeting the phosphorus requirements of broilers through inorganic P supplementation without considering the phytate phosphorus in feed ingredients can lead to increased P emissions (Lim et al., 2024). An NPP requirement of 0.39% has been reported for broiler chicks fed a conventional corn-soybean meal diet from 1 to 21 d of age (Liu et al., 2017).

Phytase is an enzyme that hydrolyzes phytate molecules, typically yielding inositol, inositol monophosphate, and inorganic phosphate (Lei et al., 2013). A meta-analysis of previous studies indicated that microbial phytase (600 FTU/kg diet) reduces the dietary NPP requirement for optimal growth performance in broilers (Faridi et al., 2015). High phytase level (12,000 U/kg diet) in a P-deficient diet has been shown to markedly increase phytate-P digestibility in broilers (Shirley and Edwards, 2003). In broilers aged from day 8 or 9 to 22 or 23, from day 3 to day 42, and from day 1 to day 50 or 56 and fed P-deficient diets (0.13% available P), supplementation with E. coli-derived phytase improves growth performance, bone development, and carcass traits (Pillai et al., 2006). The NPP relative equivalence values in birds fed 500 and 1,000 FTU phytase/kg were 0.117% and 0.166% based on ash percentage, respectively (Li et al., 2015). Recent studies have suggested that high-dose phytase supplementation can substantially reduce or even eliminate the need for supplemental inorganic P in broiler diets. For example, supplementation with 4,000 FTU/kg phytase maintained normal growth performance in broilers fed diets without inorganic P during the grower and finisher phases (Ribeiro et al., 2019). Likewise, phytase improved the performance of broilers fed NPP-deficient diets in a dose-dependent manner, with greater responses observed at ≥4,000 FTU/kg levels (Farhadi et al., 2017). Furthermore, maximal performance was achieved in broilers fed a total P-deficient corn-soybean meal diet supplemented with 12,000 FTU/kg phytase (Shirley and Edwards, 2003). Hence, the optimal phytase supplementation levels in broiler diet needs to be further elucidated, especially in diets without inorganic P addition.

Supplementation with a novel consensus phytase variant (1,000–3,000 FTU/kg) in plant-based diets without added inorganic phosphate has been shown to maintain normal growth performance in broilers throughout the production cycle (Marchal et al., 2021). Such phytase supplementation has been reported to improve ileal digestibility of nitrogen, amino acids, and energy alongside P retention and tibia ash content (Dersjant-Li et al., 2022), and maintain growth performance with a diet highly deficient in minerals, digestible amino acids, and energy in broilers (Sobotik et al., 2024). Phytase supplementation can also improve nutrient digestibility, P retention, and bone mineralization in P-deficient diets (Dersjant-Li et al., 2022; Sobotik et al., 2024). However, phytase efficacy may be affected by dietary factors such as limestone solubility, and higher supplementation levels may be required to meet digestible P requirements when highly soluble limestone is used (Bello et al., 2022). In poultry, P metabolism involves multiple organs, including the intestine, bone, and kidney. For example, intestinal P transporters such as NPT2b exhibit age-dependent expression in chickens (Wang et al., 2022). Intestinal P absorption and renal P reabsorption both adapt to diets low in available P in chickens (Li et al., 2018). However, the coordinated roles of these organs in broilers fed a non-inorganic P diets with high-dose phytase remain unclear. Moreover, whether different commercial phytase products exhibit consistent efficacy under non-inorganic P diets remains to be elucidated.

The present study aimed to evaluate the effects of two commercial phytase products supplemented at a standard (1,000 FTU/kg) or high (10,000 FTU/kg) dose on growth performance and Ca and P metabolism in broilers fed diets without supplemental inorganic P.

## 2 Materials and methods

All animal procedures were approved by the Institutional Animal Care and Use Committee of Shandong Agricultural University (SDAUA-2022-11) and were conducted in accordance with the “Guidelines for Experimental Animals” outlined by the Ministry of Science and Technology (Beijing, P. R. China).

### 2.1 Animal husbandry

A total of 600 one-day-old male Arbor Acres broilers were obtained from a local commercial hatchery. Broilers were randomly assigned to five groups, with eight replicates cages (150 × 70 × 50 cm, length × width × height) and 15 birds per cage. Each cage was equipped with a trough feeder and nipple drinkers. Broilers were brooded at 32 ± 1 °C and 55%–60% relative humidity for the first 3 days, after which the temperature was gradually reduced to 21 ± 1 °C by day 21, and maintained until day 42. Broilers were provided with 23 h of light and 1 h of darkness (23L:1D) throughout the experimental period (Sun et al., 2020). All the chickens had free access to feed and water.

### 2.2 Experimental design and diets

Two commercial phytase products widely used in the Chinese broiler industry were used in this study: P1 (RONOZYME^R^ ProAct CT, 20,000 FTU/g; DSM Co, Ltd., Shanghai, China) and P2 (HPS-20,000, 10,000 FTU/g, GBW Group Co., Ltd., Qingdao, China). Broilers were randomly assigned to 5 dietary treatments: (i) a basal diet (control) or (ii) a non-inorganic P diet supplemented with two phytases products (P1, P2) at 1,000 FTU/kg (NP1, NP2; normal level of phytase) or 10,000 FTU/kg (HP1, HP2; high level of phytase). Each treatment comprised 8 replicates, with each cage serving as a single replicate.

The basal diets were formulated with reference to the Feeding Standard of Chicken (NY/T 33-2004, China) (Ministry of Agriculture, P. R. China, 2004), using a two-phase feeding program of 1–21 and 22–42 d in accordance with the age divisions specified in this standard. The total phosphorus (TP) content of the diets was as follows: basal diet (1–21 days: 0.87% TP and 0.24% phytate P; 22–42 days: 0.84% TP and 0.22% phytate P) and non-inorganic P diet (1–21 days: 0.45% TP and 0.23% phytate P; 22–42 days: 0.42% TP and 0.22% phytate P). The ingredient compositions and nutrient levels of the experimental diet are listed in **Table 1**. Feeds were given in mash. Throughout the whole experimental period, BW were measured at 21 and 42 days of age. Bird mortality was recorded and used to calculate the mortality corrected average feed intake (FI) and feed conversion ratio (FCR).

**Table 1:** The composition and nutrient levels of the experimental diets of broilers (air–dry basis, %).

Ingredients, g/kg	1–21 days		22–42 days	
	Control	Non-inorganic P diet	Control	Non-inorganic P diet
Corn, 8.5% CP	563.10	576.20	595.20	606.50
Soybean meal, 43% CP	360.10	357.90	319.40	317.70
Soybean oil	37.30	33.10	47.50	43.90
Limestone	11.90	22.10	13.50	22.50
Calcium hydrogen phosphate	16.90	–	15.00	–
Sodium chloride	2.90	2.90	2.80	2.80
Lysine-HCl, 99%	1.00	1.00	1.00	1.00
DL-Methionine, 98%	1.60	1.60	1.10	1.10
Choline chloride, 50%	2.60	2.60	2.00	2.00
Vitamin premix^1^	0.50	0.50	0.50	0.50
Mineral premix^2^	2.00	2.00	2.00	2.00
Nutrient composition, %				
Metabolic energy^3^, MJ/kg	12.56	12.56	12.98	12.98
Crude protein^4^	21.88	22.26	19.38	19.86
Calcium^3^	0.95	0.92	0.95	0.92
Total phosphorus^3^	0.68	0.38	0.63	0.36
Available phosphorus^3^	0.39	0.09	0.35	0.08
Calcium^4^	0.85	0.80	0.88	0.84
Total phosphorus^4^	0.61	0.34	0.57	0.32
Phytate phosphorus^4^	0.24	0.23	0.22	0.22
Lysine^4^	1.06	1.06	0.97	0.97
Methionine^4^	0.37	0.37	0.31	0.31
Met+Cys^4^	0.50	0.51	0.43	0.43
Threonine^4^	1.02	1.02	0.94	0.94
Arginine^4^	1.24	1.24	1.13	1.13
Dig-Lys^3^	0.93	0.92	0.84	0.84
Dig-Met^3^	0.35	0.35	0.29	0.29
Dig-Met+Cys^3^	0.47	0.47	0.40	0.40
Dig-Thr^3^	0.91	0.91	0.86	0.86
Dig-Arg^3^	1.10	1.10	1.01	1.01

1 Vitamin premix provides the following per kg of diet: retinyl acetate, 20,000 IU; cholecalciferol, 5,500 IU; DL-α-tocopherol, 47.5 IU; menadione, 1.50 mg; thiamine, 3.41mg; riboflavin, 12.50 mg; D-pantothenic acid, 16.67 mg; niacin, 53.03 mg; pyridoxine-HCl, 6.56 mg; biotin, 0.28 mg; folic acid, 0.87 mg; yanocobalamin, 1.50 mg.

2 Mineral premix provides the following per kg of diet: Fe (as ferrous sulfate), 518.24 mg; Zn (as zinc sulfate), 278.26 mg; Mn (as manganese sulfate), 376.77 mg; Cu (as copper sulfate) 31.57 mg, I (as potassium iodide), 0.92 mg; and Se (as sodium selenite), 0.66 mg.

3 Calculated values according to China Feed Database (2020).

4 Measured values.

### 2.3 Sample collection

On days 7, 21, and 42, one chicken with BW close to the group mean was selected from each replicate, with 8 chickens per treatment group. After 12 h feed withdrawal, a blood sample was collected from the wing vein of each chicken using a negative-pressure collection vessel. The blood samples were centrifuged at 3,000 × *g* for 10 min at 4 °C to obtain serum, which was stored at –20 °C until further analysis. The birds were euthanized via cervical dislocation. Left femur and tibia were collected, cleaned of adhering tissue, and weighed. Bone length was determined using a ruler, and bone width was measured at the midpoint using a digital vernier caliper (Orientools Industrial Co., Ltd, Shanghai, China). Subsequently, the left tibia and femur were used to measure the mineral content. Tissue samples from the kidney, duodenum, and right tibia were snap-frozen in liquid nitrogen and stored at –80 °C for gene expression analysis.

### 2.4 Calcium and P retention

On days 16 and 37, one broiler close to the mean BW was selected from each replicate, with eight broilers in each treatment group. The broilers were individually reared in single cages (57 ×37 ×47 cm, length × width × height). FI was recorded daily. After 3-day of adaptation, all excreta were completely collected for three consecutive days (days 19–21 and 40–42, respectively), according to previously described methods (Thorsen et al., 2021; Dersjant-Li et al., 2022). After collection, the excreta were mixed, dried at 65 °C, and stored until further analysis.

The Ca and P retentions were calculated as follows:


Ca or P retention = (Feed intake × dietary Ca or P level) – (amount of excreta × excreta Ca or P level)

The apparent Ca and P retention rate (%) was calculated as follows:


Apparent Ca or P retention (%) = Ca or P retention/Ca or P intake × 100

### 2.5 Serum hormones and biochemical parameters

Serum concentrations of Ca and P were measured using commercial kits (Jiancheng Bioengineering Institute, Nanjing, China) and quantified by a biochemical analyzer (Hitachi L-7020, Tokyo, Japan). The activity of bone-specific alkaline phosphatase (BALP) and the levels of osteocalcin, N-terminal propeptide of type I procollagen (PINP), procollagen type I carboxy-terminal propeptide (PICP), and tartrate resistant acid phosphatase 5b (TRAP-5b) were measured using commercial ELISA kits (Shanghai Enzyme linked Biotechnology Co., Ltd., Shanghai, China) and analyzed using an ELISA reader (ELX808, BioTek, America).

The serum concentrations of parathyroid hormone (PTH) and calcitonin were measured using ELISA kits (Shanghai Enzyme-linked Biotechnology Co., Ltd., Shanghai, China). For the measurements of PTH and calcitonin, the intra- and inter-assay variation were 10% and 15%, respectively, with the lowest detectable concentrations being 3.75 pg/mL and 1 pg/mL, respectively. All samples were included in the same assay to avoid inter-assay variability.

### 2.6 Bone mineral content measurement

Bone mineral content (BMC) and bone mineral density (BMD) were measured using the dual-energy X-ray absorptiometry method (InAlyzer, Medikors LAB., Seoul, Korea). Bone bending strength was analyzed using a three-point bending test according to the method described by Song et al. (2020). The tests were performed using a microcomputer-controlled electronic universal testing machine (Jinan Shi Jin Neng, Jinan, China).

### 2.7 Chemical analysis

The experimental diets were analyzed for dry matter (method 930.15) and crude protein (CP; method 990.03) base on AOAC (2019) procedures. The Ca and P contents in the experimental diets and excreta were determined according to the method described by Li et al. (2024). Dietary AA were determined by ion-exchange chromatography with postcolumn ninhydrin detection using a Hitachi L-8900 AA Analyzer (Tokyo, Japan) after acid hydrolysis with 6 mol/L HCl and reflux for 24 h. Methionine and cysteine were analyzed as Met sulfone and cysteic acid after cold performic acid oxidation overnight before hydrolysis.

The bones were subjected to fat extraction using a 2:1 (vol/vol) ethanol: benzene mixture for 96 h, followed by drying at 60 °C for 24 h. They were then oven-dried at 105 °C for an additional 24 h and weighed to obtain the fat-free dry weight. The defatted bones were placed in a muffle furnace at 550 °C to determine the ash content. The ash percentage was calculated and expressed as a percentage of the dry-defatted bone weight. Bone ash concentration was expressed as ash weight per unit bone volume. Bone Ca and P contents were determined as described above.

### 2.8 RNA extraction and real-time quantitative PCR

RNA extraction and real-time PCR were conducted according to the method described by Song et al. (2020) and Li et al. (2024). Total RNA was extracted from the duodenum, kidneys, and tibia using TransZol Up reagent (TransGen Biotech). RNA concentration and purity were verified from OD260/280 readings (1.75–2.01) using spectrophotometry (Eppendorf, Germany). Reverse transcription was performed using total RNA (1 μg) for first-strand cDNA synthesis with a Transcriptor First-Strand cDNA Synthesis Kit (Roche, Germany). The cDNA was amplified in a 20 μL PCR system containing 0.2 μmol/L of each specific primer (Sangon, China) and SYBR Green master mix (Roche, Germany) according to the manufacturer’s instructions. Real-time quantitative PCR was performed under the following cycling conditions: 95 °C for 30 s, followed by 40 cycles of 95 °C for 5 s and 60 °C for 30 s (Applied Biosystems, Thermo Fisher Scientific, USA). The mRNA levels of Calbindin-D28k (*CaBP-D28k*), fibroblast growth factor 23 (*FGF23*), Na^+^-dependent phosphate cotransporter 2a (*NPt2a*), Na^+^-dependent phosphate cotransporter-2b (*NPt2b*), and plasma membrane Ca-ATPase 1b (*PMCA-1b*) were determined. The primer sequences are listed in **Table 2**. The primer sequences for each gene were synthesized by Shanghai Shenggong Bioengineering Co., Ltd. Gene sequencing confirmed that the PCR products of each gene shared >99% with original gene sequences. Data were analyzed using the 2^−ΔΔCt^ method (Livak and Schmittgen, 2001).

**Table 2:** Primers for the targeted and reference transcripts.

Gene	GeneBank accession no.	Product length (bp)	Primer sequences
*CaBP-D28k*	NC_006089.4	132	F: TGTTATGGAGTGCAGGATGG
			R: TAGAGCGAACAAGCAGGTGA
*FGF23*	XM_425663.4	203	F: CCTTGGGACAGAAACTGCTC
			R: CACCAATTTCAAAAGGAACG
*NPt2a*	NC_006100.4	249	F: CCAAACTGCACGGCTTCT
			R: TGGGAGGTCAGTGTTGATGA
*NPt2b*	NC_006091.4	113	F: ACTGGCTTGCTGTGTTTGC
			R: AGGGGCATCTTCACCACTTT
*PMCA-1b*	NC_006106.4	98	F: TTCAGGTACTCATGTGATGGAAGG
			R: CAGCCCCAAGCAAGGTAAAG
*α-Klotho*	NC_052573.1	184	F: AATGGCACAGCACCCGTCAATC
			R: CAGCGTAGTCGTGGAAGAGGTTG
*β-actin*	L08165	300	F:TGCGTGACATCAAGGAGAAG
			R:TGCCAGGGTACATTGTGGTA

*CaBP-D28k*, Calbindin-D28k; *FGF23*, fibroblast growth factor 23; *NPt2a*, Na^+^-dependent phosphate cotransporter 2a; *NPt2b*, Na^+^-dependent phosphate cotransporter 2b; *PMCA-1b*, plasma membrane Ca^2+^-ATPase 1b.

### 2.9 Statistical analysis

All the data were examined for homogeneity and normality of variances among the treatments using the UNIVARIATE procedure. The main effect of dietary treatment was evaluated using one-way ANOVA and different phytase treatments were compared with control group (SAS Version 8e, SAS Institute, Cary, NC, USA). The main effects of phytase source and level and their interactions were estimated using two-way ANOVA. Tukey’s honest significant difference test was used for the *post hoc* analysis of means. Differences were considered statistically significant at *p* < 0.05.

## 3 Results

### 3.1 Growth performance

In the starter phase (1–21 days of age), dietary treatment significantly influenced FI and BW gain (*p* < 0.001; **Table 3**). FI decreased in NP1 (*p* < 0.01; **Table 3**), whereas BW gain decreased in NP1 and NP2 (*p* < 0.01), compared with that in the control group. By contrast, FI and BW gain were not influenced by the HP1 and HP2 treatments (*p* > 0.05). Both phytase sources and level had significant effects on FI (*p <* 0.05) and BW gain (*p* < 0.001). The P2 and HP treatments had higher FI and BW gain than the P1 and NP treatments (*p* < 0.05). There was no interaction of phytase source and level on FI and BW gain (*p* > 0.05). By contrast, FCR was not significantly altered by dietary treatment and phytase source or level (*p* > 0.05).

**Table 3:** Effects of phytase sources and supplemental levels on the growth performance of broilers fed a diet with non-inorganic phosphate supplementation.

	Dietary treatment^1^							Phytase effect^2^						
	Control	NP1	HP1	NP2	HP2	SEM	*p* value	P1	P2	NP	HP	*p* value		
												Source	Level	Source × level
1–21 days														
FI (g/d)	30.0^ab^	26.8^c^	29.6^ab^	28.2^bc^	31.4^a^	0.38	<0.001	28.2^y^	29.8^x^	27.5^n^	30.5^m^	0.035	s<0.001	0.727
BW gain (g/d)	22.0^a^	18.9^b^	22.0^a^	20.3^b^	23.2^a^	0.34	<0.001	20.4^y^	21.8^x^	19.6^n^	22.6^m^	0.023	<0.001	0.847
FCR (g/g)	1.36	1.43	1.35	1.39	1.35	0.02	0.474	1.39	1.37	1.41	1.35	0.586	0.116	0.476
22–42 days														
FI (g/d)	116.1^a^	105.7^b^	114.9^a^	102.6^b^	117.6^a^	0.68	<0.001	110.3	110.1	104.2^n^	116.2^m^	0.884	<0.001	0.068
BW gain (g/d)	61.7^b^	49.6^d^	66.2^a^	55.6^c^	64.8^ab^	0.67	<0.001	57.9	60.2	52.6^n^	65.5^m^	0.080	<0.001	0.007
FCR (g/g)	1.88^b^	2.14^a^	1.74^c^	1.85^bc^	1.82^bc^	0.02	<0.001	1.94^x^	1.83^y^	2.00^n^	1.78^m^	0.019	<0.001	<0.001
1–42 days														
FI (g/d)	70.2^a^	63.7^b^	69.4^a^	63.2^b^	71.9^a^	1.21	<0.001	66.6	67.6	63.5^n^	70.6^m^	0.250	<0.001	0.086
BW gain (g/d)	41.5^a^	32.6^c^	41.9^a^	36.3^b^	42.1^a^	1.12	<0.001	37.2^y^	39.2^x^	34.4^n^	42.0^m^	0.003	<0.001	0.007
FCR (g/g)	1.69^b^	1.96^a^	1.66^b^	1.75^b^	1.71^b^	0.03	<0.001	1.81^y^	1.72^x^	1.85^n^	1.68^m^	0.005	<0.001	<0.001

NP1, 1,000FTU/kg phytase 1; HP1, 10,000FTU/kg phytase 1; NP2, 1,000FTU/kg phytase 2; and HP2, 10,000FTU/kg phytase 2.

1 Effect of dietary treatments was evaluated with one-way ANOVA (n=8). ^2^Effects of phytase sources and supplemental levels and their interaction were evaluated with two-way ANOVA. ^a,b^Means with different superscripts in the same row differ significantly (*p* < 0.05). ^m,n^Means with different superscripts between phytase sources differ significantly (*p* < 0.05). ^x,y^Means with different superscripts between phytase levels differ significantly (*p* < 0.05).

From 22 to 42 days of age, FI, BW gain, and FCR were significantly affected by dietary treatment (*p* < 0.001). Compared with the control group, NP1 and NP2 decreased FI (*p* < 0.05), whereas HP1 and HP2 had no effect (*p* > 0.05). By contrast, BW gain decreased (*p* < 0.05) in NP1 and NP2, remained unchanged in HP2 (*p* > 0.05), and increased in HP1 (*p* < 0.05). Compared with the control group, NP1 increased FCR, whereas HP1 decreased FCR, while NP2 and HP2 did not significantly affect FCR. Phytase level had a significant influence (*p* < 0.001) on FI, and HP had higher FI than NP. A significant interaction was observed between phytase source and level for BW gain (*p* < 0.01) and FCR (*p* < 0.001). HP1 had the highest BW gain and the lowest FCR, whereas NP1 had the lowest BW gain and highest FCR.

Throughout the growth period (1–42 days of age), dietary treatment had a significant effect (*p* < 0.001) on FI, BW gain, and FCR. Compared with the control group, FI and BW gain were decreased by NP1 and NP2 (*p <* 0.01), whereas were not influenced by HP1 and HP2 (*p* > 0.05). FCR value was increased by NP1 but remained unchanged in NP2, HP1, and HP2. A significant interaction was observed between phytase source and level for BW gain (*p* < 0.01) and FCR (*p* < 0.001). Compared to other treatments, NP1 had the lowest BW gain and the highest FCR.

### 3.2 Calcium and P metabolism

During the starter phase (days 19–21), dietary treatment, phytase source, and supplementation level had no significant effects on Ca intake, excretal Ca, Ca excretion, Ca retention, or apparent Ca retention (*p* > 0.05; **Table 4**). Compared with the control group, P intake, excretal P, P excretion, and P retention were significantly decreased in all phytase-supplemented groups (*p* < 0.001), whereas dietary treatment tended to affect apparent P retention (*p* = 0.068), with numerically higher values in the phytase-supplemented groups. Phytase supplementation level significantly affected P retention, with a greater value in HP than in NP broilers (*p* = 0.021), but had no significant effect on apparent P retention (*p* = 0.170). Phytase source had no significant effect on P retention or apparent P retention (*p* > 0.05), and no source × level interaction was detected for these variables (*p* > 0.05).

**Table 4:** Effects of phytase source and supplemental level on calcium (Ca) and phosphorus (P) balance of broilers fed a diet with non-inorganic phosphate supplementation in the period of 19–21 days of age (Dry matter basis).

	Dietary treatment ^1^							Phytase effect ^2^						
	Control	NP1	HP1	NP2	HP2	SEM	*p* value	P1	P2	NP	HP	*p* value		
												Source	Level	Source × level
Feed intake (g/day)	58.9	56.9	63.8	60.7	63.1	0.91	0.069	60.4	61.9	58.8^n^	63.5^m^	0.403	0.017	0.216
Excreta output (g/day)	12.2^ab^	10.8^b^	13.7^a^	13.3^a^	13.5^a^	0.32	0.005	12.3^y^	13.4^x^	12.1^n^	13.6^m^	0.041	0.007	0.018
Ca intake (g/day)	0.50	0.45	0.50	0.52	0.48	0.01	0.257	0.47	0.50	0.48	0.49	0.297	0.791	0.053
Excretal Ca (%)	2.20	2.28	1.97	2.20	2.03	0.07	0.675	2.13	2.12	2.24	2.00	0.962	0.172	0.706
Ca excretion (g/day)	0.27	0.25	0.27	0.29	0.27	0.01	0.603	0.26	0.28	0.27	0.27	0.284	0.918	0.231
Ca retention (g/day)	0.23	0.20	0.23	0.22	0.21	0.01	0.859	0.22	0.22	0.21	0.22	0.995	0.712	0.382
Apparent Ca retention (%)	46.2	45.2	45.9	42.6	43.4	1.59	0.954	45.6	43.0	43.9	44.7	0.523	0.851	0.986
P intake (g/day)	0.51^a^	0.25^b^	0.28^b^	0.28^b^	0.28^b^	0.02	<0.001	0.27	0.28	0.26	0.28	0.302	0.077	0.296
Excretal P (%)	1.32^a^	0.59^b^	0.51^b^	0.58^b^	0.49^b^	0.07	<0.001	0.55	0.53	0.58	0.50	0.749	0.092	0.957
P excretion (g/day)	0.16^a^	0.06^b^	0.07^b^	0.08^b^	0.07^b^	0.01	<0.001	0.07	0.07	0.07	0.07	0.465	0.678	0.245
P retention (g/day)	0.35^a^	0.19^c^	0.22^bc^	0.20^bc^	0.22^b^	0.01	<0.001	0.20	0.21	0.19^n^	0.22^m^	0.488	0.021	0.708
Apparent P retention (%)	68.6	74.8	75.6	72.0	76.9	1.05	0.068	75.2	74.5	73.4	76.3	0.726	0.170	0.316

NP1, 1,000FTU/kg phytase 1; HP1, 10,000FTU/kg phytase 1; NP2, 1,000FTU/kg phytase 2; and HP2, 10,000FTU/kg phytase 2. ^1^Effect of dietary treatments was evaluated with one-way ANOVA (n=8). ^2^Effects of phytase sources and supplemental levels and their interaction were evaluated with two-way ANOVA. ^a,b^Means without a common superscript in the same row differ significantly (*p* < 0.05). ^m,n^Means with different superscripts between phytase sources differ significantly (*p* < 0.05). ^x,y^Means with different superscripts between phytase levels differ significantly (*p* < 0.05).

In the finisher phase (days 40–42), FI (*p* < 0.001), Ca intake (*p* < 0.01), and Ca retention (*p* < 0.05) were significantly lower in NP1 than in the control group (**Table 5**). In contrast, excretal Ca, Ca excretion, and apparent Ca retention were not significantly affected by dietary treatment (*p* > 0.05). Compared with the control group, P intake, excretal P, P excretion, and P retention were significantly decreased in all phytase-supplemented groups (*p* < 0.001), whereas apparent P retention was significantly increased at both supplementation levels (1,000 and 10,000 FTU/kg; *p* < 0.001). However, apparent P retention did not differ between the two supplementation levels (*p* = 0.218). Both phytase source and supplementation level significantly affected Ca intake and Ca retention (*p* < 0.05), with greater values at P2 than at P1 and with HP than with NP. In contrast, P intake, excretal P, P excretion, P retention, and apparent P retention were not significantly affected by phytase source or supplementation level (*p* > 0.05). No significant source × level interactions were detected (*p* > 0.05).

**Table 5:** Effects of phytase source and supplemental level on calcium (Ca) and phosphorus (P) balance of broilers fed a diet with non-inorganic phosphate supplementation in the period of 40–42 days of age (Dry matter basis).

	Dietary treatment^1^							Phytase effect^2^						
	Control	NP1	HP1	NP2	HP2	SEM	Diet	P1	P2	NP	HP	*p* value		
												Source	Level	Source × level
Feed intake (g/day)	184.9^a^	151.6^b^	171.3^ab^	176.6^a^	180.0^a^	3.11	<0.001	161.5^y^	178.3^x^	164.1^n^	175.7^m^	0.001	0.014	0.070
Excreta output (g/day)	31.8^a^	26.0^b^	27.9^ab^	30.0^ab^	28.2^ab^	0.69	0.060	27.0	29.1	28.0	28.0	0.121	0.980	0.169
Ca intake (g/day)	1.62^a^	1.26^b^	1.43^ab^	1.44^ab^	1.55^a^	0.04	0.006	1.35^y^	1.49^x^	1.35^n^	1.49^m^	0.027	0.026	0.566
Excretal Ca (%)	2.25	2.16	2.07	2.04	2.16	0.06	0.864	2.12	2.10	2.10	2.12	0.880	0.911	0.477
Ca excretion (g/day)	0.71^a^	0.56^b^	0.58^b^	0.61^ab^	0.60^ab^	0.02	0.061	0.57	0.61	0.59	0.59	0.285	0.907	0.782
Ca retention (g/day)	0.91^a^	0.70^b^	0.86^a^	0.83^ab^	0.94^a^	0.03	0.017	0.78^y^	0.88^x^	0.76^n^	0.90^m^	0.040	0.008	0.605
Apparent Ca retention (%)	56.0	55.3	59.9	57.6	60.9	0.94	0.273	57.6	59.2	56.4	60.4	0.426	0.070	0.764
P intake (g/day)	1.55^a^	0.65^b^	0.71^b^	0.73^b^	0.77^b^	0.08	<0.001	0.68	0.75	0.69	0.74	0.125	0.254	0.789
Excretal P (%)	1.63^a^	0.59^b^	0.55^b^	0.52^b^	0.55^b^	0.10	<0.001	0.57	0.54	0.55	0.55	0.260	0.923	0.321
P excretion (g/day)	0.52^a^	0.15^b^	0.15^b^	0.16^b^	0.15^b^	0.03	<0.001	0.15	0.16	0.15	0.15	0.794	0.989	0.735
P retention (g/day)	1.03^a^	0.49^b^	0.55^b^	0.57^b^	0.62^b^	0.05	<0.001	0.52	0.59	0.53	0.59	0.122	0.236	0.829
Apparent P retention (%)	66.3^b^	76.4^a^	78.2^a^	78.4^a^	79.8^a^	1.23	<0.001	77.3	79.1	77.4	79.0	0.176	0.218	0.853

NP1, 1,000FTU/kg phytase 1; HP1, 10,000FTU/kg phytase 1; NP2, 1,000FTU/kg phytase 2; and HP2, 10,000FTU/kg phytase 2. ^1^Effect of dietary treatments was evaluated with one-way ANOVA (n=8). ^2^Effects of phytase sources and supplemental levels and their interaction were evaluated with two-way ANOVA. ^a,b^Means without same superscript in the same row differ significantly (*p* < 0.05). ^m,n^Means with different superscripts between phytase sources differ significantly (*p* < 0.05). ^x,y^Means with different superscripts between phytase levels differ significantly (*p* < 0.05).

### 3.3 Serum hormones and metabolites

Dietary treatment had a significant influence (*p <* 0.05) on serum Ca levels on days 7, 21, and 42 (**Table 6**). Compared with the control group, NP1 increased serum Ca levels on day 7, and NP1, NP2, and HP2 increased serum Ca levels on day 42. Serum Ca was influenced (*p* < 0.01) by phytase source on day 21 and by phytase level on day 42. The interaction of phytase source and level influenced serum Ca levels on day 7 (*p* < 0.001) and day 42 (*p* < 0.05). In P1 treatment, serum Ca was lower (*p* < 0.05) in HP than that in NP, whereas there was no difference (*p* > 0.05) between NP and HP in P2 treatment. Serum P levels were significantly affected by dietary treatment on days 21 and 42 (*p* < 0.01). Compared with the control group, serum P levels were decreased in NP1 and NP2 on days 21 and 42 (*p <* 0.01). Phytase source changed serum P on day 42 and P2 had higher serum P than that in P1 (*p* < 0.05). By contrast, serum P was increased by HP on days 21 and 42 (*p* < 0.01). A significant interaction was observed between phytase source and level for serum P levels (*p* < 0.01) and HP1 had the highest serum P level, whereas NP1 had the lowest (**Table 6**).

**Table 6:** Effects of phytase sources and levels on serum calcium (Ca), phosphorus (P), parathyroid hormone (PTH) and calcitonin of broilers fed a diet with non-inorganic phosphorus supplementation.

	Day of age	Dietary treatment^1^							Phytase effect^2^						
		Control	NP1	HP1	NP2	HP2	SEM	*p* value	P1	P2	NP	HP	*p v*alue		
													Source	Level	Source × level
Ca (mmol/L)	D 7	2.80^b^	3.22^a^	2.54^b^	2.63^b^	2.91^ab^	0.06	0.003	2.88	2.75	2.92	2.71	0.282	0.060	s<0.001
	D 21	3.11^ab^	3.15^ab^	2.97^b^	3.45^a^	3.47^a^	0.06	0.016	3.06^y^	3.46^x^	3.30	3.22	0.004	0.534	0.426
	D 42	2.99^b^	3.47^a^	2.79^b^	3.38^a^	3.32^a^	0.05	<0.001	3.23	3.35	3.42^m^	3.06^n^	0.146	0.003	0.018
P (mmol/L)	D 7	1.24	0.99	1.03	1.08	1.06	0.05	0.636	1.01	1.07	1.03	1.05	0.578	0.901	0.799
	D 21	1.87^a^	1.31^b^	1.56^ab^	1.34^b^	1.80^a^	0.06	0.002	1.44	1.57	1.32^n^	1.68^m^	0.245	0.003	0.362
	D 42	2.08^a^	1.34^c^	2.05^a^	1.79^b^	2.01^ab^	0.06	<0.001	1.69^y^	1.90^x^	1.56^n^	2.03^m^	0.016	<0.001	0.005
PTH (ng/mL)	D 7	83.8	83.0	84.1	75.4	79.2	1.89	0.549	83.6	77.3	79.2	81.7	0.195	0.606	0.779
	D 21	94.0^a^	75.4^c^	86.9^ab^	79.6^bc^	92.5^a^	1.97	0.005	81.2	86.0	77.5^n^	89.7^m^	0.240	0.006	0.861
	D 42	96.8	89.7	95.7	94.2	96.2	0.93	0.101	92.7	95.2	92.0^n^	96.0^m^	0.186	0.041	0.294
Calcitonin (ng/mL)	D 7	24.9^a^	24.2^ab^	23.0^ab^	22.3^c^	21.8^c^	0.29	0.001	23.9^x^	22.1^y^	23.6^m^	22.4^n^	0.001	0.030	0.196
	D 21	22.6	22.7	22.5	23.8	24.8	0.31	0.090	22.6^y^	24.9^x^	23.3	23.6	0.015	0.601	0.372
	D 42	30.1	29.2	28.8	29.5	29.4	0.38	0.904	29.4	29.4	29.8	29.1	0.991	0.394	0.513

NP1, 1,000 FTU/kg phytase 1; HP1, 10,000 FTU/kg phytase 1; NP2, 1,000 FTU/kg phytase 2; and HP2, 10,000 FTU/kg phytase 2.

1 Effect of dietary treatments was evaluated with one-way ANOVA (n=8). ^2^Effects of phytase sources and supplemental levels and their interaction were evaluated with two-way ANOVA. ^a,b^Means without a common superscript in the same row differ significantly (*p* < 0.05). ^m,n^Means with different superscripts between phytase sources differ significantly (*p* < 0.05). ^x,y^Means with different superscripts between phytase levels differ significantly (*p* < 0.05).

Compared with the control group, NP1 and NP2 significantly decreased PTH levels day 21 (*p <* 0.01; **Table 6**). Compared with the NP treatment, the HP treatment increased PTH levels on days 21 (*p <* 0.01) and 42 (*p <* 0.05). Serum calcitonin levels were significantly affected by dietary treatment and phytase source and level on day 7 (*p <* 0.01). Calcitonin levels in HP1 and HP2 groups were lower than those in the control group. On day 7, P2 and NP had higher calcitonin levels (*p* < 0.01) than P1 and HP.

BALP levels were not significantly affected (*p* > 0.05) by dietary treatment on days 7, 21 and 42 (**Table 7**). However, on day 21, the HP treatment significantly (*p* < 0.05) decreased BALP levels compared with the NP treatment. Dietary treatment significantly altered (*p* < 0.05) osteocalcin levels on day 21. The osteocalcin levels in the NP2 group were lower than those in the control group. Moreover, osteocalcin levels were higher in the HP group (*p* < 0.05) than in the NP group on day 21. Compared to the control, serum PINP concentrations were significantly decreased by all phytase treatments (*p* < 0.01) on day 42, with HP treatment showing lower PINP levels than the NP treatment (*p* < 0.05).

**Table 7:** Effects of phytase sources and levels on bone formation markers of broilers fed a diet with non-inorganic phosphorus supplementation (ng/mL).

	Day of age	Dietary treatment^1^							Phytase effect^2^						
		Control	NP1	HP1	NP2	HP2	SEM	*p* value	P1	P2	NP	HP	*p* value		
													Source	Level	Source × level^2^
BALP	D 7	1690	1700	1715	1697	1714	11.73	0.960	1707	1705	1698	1714	0.952	0.572	0.971
	D 21	1752	1801	1705	1852	1733	11.73	0.083	1753	1792	1827^m^	1719^n^	0.346	0.014	0.781
	D 42	1771	1864	1776	1807	1759	18.48	0.280	1811	1783	1802	1792	0.367	0.728	0.063
Osteocalcin	D 7	8.62	7.96	8.03	7.95	7.86	0.11	0.215	7.99	7.90	7.95	7.95	0.719	0.988	0.763
	D 21	8.79^a^	7.98^ab^	8.18^ab^	7.60^b^	8.79^a^	0.14	0.015	8.08	8.19	7.79^n^	8.48^m^	0.692	0.019	0.087
	D 42	12.0	11.8	11.2	10.7	10.9	0.20	0.162	11.5	10.8	11.2	11.0	0.086	0.520	0.312
PINP	D 7	29.1	31.8	31.3	28.8	30.1	0.81	0.724	31.6	29.4	30.3	30.7	0.207	0.819	0.586
	D 21	27.5	28.3	26.9	32.0	29.0	0.86	0.388	27.6	30.5	30.2	28.0	0.139	0.265	0.667
	D 42	32.2^a^	25.9^b^	24.3^bc^	25.3^bc^	23.0^c^	0.64	<0.001	25.1	24.1	25.6^m^	23.6^n^	0.287	0.037	0.726
PICP	D 7	14.8^a^	13.8^ab^	13.3^bc^	12.5^c^	12.9^bc^	0.19	0.001	13.6^x^	12.8^y^	13.2	13.2	0.023	0.985	0.173
	D 21	12.9	12.5	12.5	12.0	12.0	0.16	0.325	12.5	12.0	12.3	12.2	0.109	0.929	0.992
	D 42	12.5	12.5	12.6	12.1	12.4	0.22	0.965	12.5	12.2	12.3	12.5	0.614	0.709	0.847
TRAP-5b	D 7	14.8^ab^	13.6^b^	14.3^ab^	15.3^a^	13.7^ab^	0.20	0.030	14.0	14.5	14.5	14.0	0.170	0.277	0.009
	D 21	14.4	13.9	14.7	13.0	14.7	0.22	0.074	14.3	13.9	13.5^n^	14.7^m^	0.409	0.010	0.334
	D 42	15.9^a^	15.8^a^	15.2^ab^	14.4^b^	15.3^ab^	0.15	0.006	15.5	14.9	15.1	15.2	0.033	0.712	0.013

NP1, 1,000 FTU/kg phytase 1; HP1, 10,000 FTU/kg phytase 1; NP2, 1,000 FTU/kg phytase 2; and HP2, 10,000 FTU/kg phytase 2.

1 Effect of dietary treatments was evaluated with one-way ANOVA (n=8). ^2^Effects of phytase sources and supplemental levels and their interaction were evaluated with two-way ANOVA. ^a,b^Means without a common superscript in the same row differ significantly (*p* < 0.05). ^m,n^Means with different superscripts between phytase sources differ significantly (*p* < 0.05). ^x,y^Means with different superscripts between phytase levels differ significantly (*p* < 0.05). BALP, bone-specific alkaline phosphatase; PINP, N-terminal propeptide of type I procollagen; PICP, procollagen type I carboxy-terminal propeptide, and TRAP-5b, tartrate resistant acid phosphatase 5b.

On day 7, PICP concentrations was significantly influenced by dietary treatment (*p* < 0.01). PICP levels were lower in the HP1, NP1, and HP2 groups than in the control group. By contrast, PICP did not change (*p* > 0.05) with the dietary treatment on day 21 or day 42. Moreover, the P1 broilers had higher PICP levels than the P2 broilers on day 7 (*p* < 0.05). Dietary treatment significantly affected (*p* < 0.05) TRAP-5b concentrations on days 7 and 42, with higher levels in the NP2 than in NP1 on day 7 and the opposite was true on day 42. On day 21, TRAP-5b level was higher in HP groups than that in the NP groups (**Table 7**).

### 3.4 Tibia bone measurements

Compared with the control tibia length, the HP2 tibia length was significantly increased on day 21, whereas the NP1 tibia length was decreased on day 42 (*p* < 0.01; **Table 8**). Both the phytase source and level had a significant effect on tibia bone length on days 21 and 42, which was increased by the P2 and HP treatments (*p* < 0.05). On day 21, phytase source altered bone width, with P2 broilers having greater bone width than the P1 broilers (*p* < 0.01). Compared with the control, the FW and FFDW of the tibia were not significantly affected by dietary treatment on days 7 and 42 (*p* > 0.05). On day 21, FFDW was higher in the P2 broilers than in the P1 broilers (*p* < 0.05). There was a significant interaction of phytase source and level (*p* < 0.05), with the highest FFDW level in HP2 than in other groups.

**Table 8:** Effects of phytase sources and levels on tibia length, width, fresh weight, fat-free dry weight of broilers fed a diet with non-inorganic phosphorus supplementation.

	Day of age	Dietary treatment^1^								Phytase effect^2^						
		Control	NP1	HP1	NP2		HP2	SEM	*p* value	P1	P2	NP	HP	*p v*alue		
														Source	Level	Source × level
Length (mm)	D 7	41.2	40.6	40.6	40.8	40.5		0.19	0.801	40.6	40.6	40.7	40.6	0.991	0.721	0.747
	D 21	59.9^b^	60.6^b^	61.1^b^	61.6^b^	63.4^a^		0.31	0.002	60.9^y^	62.5^x^	61.1^n^	62.3^m^	0.009	0.050	0.276
	D 42	99.5^a^	94.0^b^	101.5^a^	99.6^a^	102.0^a^		0.73	0.002	97.8^y^	100.8^x^	96.8^n^	101.8^m^	0.050	0.002	0.090
Width (mm)	D 7	2.19	2.16	2.12	2.16	2.00		0.03	0.241	2.14	2.08	2.16	2.06	0.321	0.130	0.331
	D 21	3.75^ab^	3.70^b^	3.68^b^	4.00^ab^	4.06^a^		0.05	0.039	3.69^y^	4.03^x^	3.85	3.87	0.002	0.860	0.682
	D 42	6.78	6.56	7.35	7.27	7.30		0.13	0.190	6.96	7.28	6.92	7.32	0.231	0.141	0.167
Fresh weight (g)	D 7	0.89	0.89	0.89	0.87	0.98		0.02	0.567	0.89	0.89	0.88	0.90	0.966	0.720	0.743
	D 21	0.88^ab^	0.92^ab^	0.83^b^	0.88^ab^	0.97^a^		0.02	0.043	0.88	0.89	0.90	0.86	0.812	0.510	0.355
	D 42	0.80	0.85	0.82	0.88	0.83		0.02	0.210	0.84	0.82	0.87	0.79	0.699	0.056	0.296
Fat-free dry weight (g)	D 7	0.25	0.23	0.24	0.24	0.25		0.004	0.333	0.23	0.24	0.23	0.25	0.199	0.132	0.950
	D 21	0.28^ab^	0.26^b^	0.25^b^	0.26^b^	0.30^a^		0.005	0.006	0.26^y^	0.28^x^	0.26	0.28	0.008	0.126	0.025
	D 42	0.30	0.30	0.27	0.31	0.31		0.006	0.252	0.29	0.31	0.30	0.29	0.087	0.272	0.384

NP1, 1,000FTU/kg phytase 1; HP1, 10,000FTU/kg phytase 1; NP2, 1,000FTU/kg phytase 2; and HP2, 10,000FTU/kg phytase 2.

1 Effect of dietary treatments was evaluated with one-way ANOVA (n=8). ^2^Effects of phytase sources and supplemental levels and their interaction were evaluated with two-way ANOVA. ^a,b^Means without a common superscript in the same row differ significantly (*p* < 0.05). ^m,n^Means with different superscripts between phytase sources differ significantly (*p* < 0.05). ^x,y^Means with different superscripts between phytase levels differ significantly (*p* < 0.05).

The BMC content was significantly influenced by dietary treatment. Compared with the control, the BMC content was decreased (*p* < 0.05) by the NP1 treatment on day 7 and increased (*p* < 0.01) by HP1 and HP2 treatments on day 42 (**Table 9**). Similarly, compared to the control group, BMD was decreased by all phytase treatments on day 7 (*p* < 0.01) and decreased by NP1 on day 21 (*p* < 0.01), whereas it was increased by HP1 and HP2 on day 42. Phytase level had a significant effect (*p* < 0.01) on BMC and BMD at days 21 and 42. The HP broilers had higher BMC levels than the NP broilers. The bone ash content decreased on day 7 in the NP1 and HP1 groups (*p* < 0.01), decreased on day 21 in the NP1 and NP2 groups, and decreased on day 42 in the NP1 group compared with that in the control group (**Table 9**). Phytase source, level, and their interaction had significant effects on days 7 and 42 (*p* < 0.01). Compared with the P1 and NP treatments, the P2 and HP treatments increased the bone ash content (*p* < 0.01). However, on day 21, only a significant effect on phytase levels was detected (*p* < 0.01).

**Table 9:** Effects of phytase sources and levels on tibial mineral contents of broilers fed a diet with non-inorganic phosphorus supplementation. .

	Day of age	Dietary treatment^1^							Phytase effect^2^						
		Control	NP1	HP1	NP2	HP2	SEM	*p* value	P1	P2	NP	HP	*p* value		
													Source	Level^2^	Source × level^2^
BMC (g)	D 7	0.06^a^	0.04^b^	0.05^ab^	0.05^ab^	0.05^ab^	0.00	0.034	0.04	0.05	0.04	0.05	0.129	0.298	0.567
	D 21	0.28^abc^	0.24^c^	0.29^ab^	0.27^bc^	0.32^a^	0.01	0.015	0.26	0.30	0.26^n^	0.31^m^	0.059	0.004	0.974
	D 42	1.27^bc^	1.13^c^	1.96^a^	1.46^b^	1.94^a^	0.07	<0.001	1.55	0.70	1.29^n^	1.95^m^	0.066	<0.001	0.034
BMD (g/cm^2^)	D 7	0.04^a^	0.02^c^	0.03^bc^	0.03^bc^	0.03^b^	0.00	<0.001	0.02	0.03	0.02	0.03	0.148	0.091	0.674
	D 21	0.06^a^	0.05^b^	0.06^a^	0.05^ab^	0.06^a^	0.00	0.008	0.05	0.05	0.05^n^	0.06^m^	0.258	0.002	0.354
	D 42	0.11^bc^	0.09^c^	0.14^a^	0.11^c^	0.14^ab^	0.01	0.001	0.12	0.12	0.10^n^	0.14^m^	0.557	<0.001	0.218
Ash (%)^3^	D 7	34.7^a^	28.8^c^	32.7^b^	33.2^ab^	34.1^ab^	0.53	<0.001	30.7^y^	33.6^x^	31.0^n^	33.4^m^	<0.001	<0.001	<0.001
	D 21	37.1^a^	34.4^b^	37.4^a^	35.2^b^	37.5^a^	0.28	<0.001	35.9	36.4	34.8^n^	37.4^m^	0.131	<0.001	0.306
	D 42	42.1^ab^	36.6^c^	43.0^a^	38.4^b^	42.1^ab^	0.40	<0.001	39.8^y^	40.3^x^	37.5^n^	42.6^m^	0.005	<0.001	<0.001
Ca (%)^3^	D 7	8.11^a^	6.39^b^	8.10^a^	6.93^b^	8.82^a^	0.24	0.001	7.24	7.88	6.66^n^	8.46^m^	0.088	<0.001	0.800
	D 21	12.0^b^	11.1^c^	13.0^a^	10.7^c^	12.1^b^	0.19	<0.001	11.4^y^	12.0^x^	10.9^n^	12.6^m^	0.008	<0.001	0.229
	D 42	15.4^b^	13.0^d^	17.3^a^	14.4^c^	17.2^a^	0.29	<0.001	15.2^y^	15.8^x^	13.7^n^	17.3^m^	0.034	<0.001	0.009
P (%)^3^	D 7	8.81	7.67	9.15	8.78	9.14	0.22	0.187	8.41	8.96	8.22	9.15	0.250	0.061	0.238
	D 21	10.1^a^	8.54^c^	10.3^a^	9.37^b^	10.3^a^	0.14	<0.001	9.43^y^	9.82^x^	8.96^n^	10.3^m^	<0.001	<0.001	<0.001
	D 42	11.1^a^	9.48^c^	11.3^a^	10.4^b^	11.1^a^	0.11	<0.001	10.4^y^	10.7^x^	9.92^n^	11.2^m^	0.001	<0.001	<0.001
Breaking strength(N)	D 7	20.9^a^	15.4^b^	14.6^b^	15.2^b^	14.6^b^	0.54	<0.001	15.0	14.9	15.3	14.6	0.950	0.491	0.950
	D 21	66.7^bc^	54.9^c^	74.1^b^	73.1^b^	90.1^a^	2.83	<0.001	64.5^y^	81.6^x^	64.0^n^	82.1^m^	0.003	0.002	0.833
	D 42	239.3^ab^	178.4^c^	285.9^a^	234.4^b^	283.0^a^	9.18	<0.001	232.1	258.7	206.4^n^	284.4^m^	0.069	<0.001	0.045

NP1, 1,000FTU/kg phytase 1; HP1, 10,000FTU/kg phytase 1; NP2, 1,000FTU/kg phytase 2; and HP2, 10,000FTU/kg phytase 2. BMC, bone mineral content; BMD, bone mineral density. ^1^Effect of dietary treatments was evaluated with one-way ANOVA (n=8). ^2^Effects of phytase sources and supplemental levels and their interaction were evaluated with two-way ANOVA. ^3^ Dry matter basis. ^a,b^Means without a common superscript in the same row differ significantly (*p* < 0.05); ^m,n^Means with different superscripts between phytase sources differ significantly (*p* < 0.05). ^x,y^Means with different superscripts between phytase levels differ significantly (*p* < 0.05).

On day 7, the Ca levels were altered (*p* < 0.01) by dietary treatment. The Ca content in the NP broilers was significantly lower than that in the control and HP broilers (**Table 9**). On day 21, Ca levels decreased in the NP1 and NP2 groups but increased in the HP1 group compared with those in the control group (*p* < 0.01). The P2 and HP treatments significantly increased Ca levels on day 21 (*p* < 0.01). On day 42, the bone Ca concentration increased in response to the P2 and HP treatments (*p* < 0.01). Ca levels decreased in the NP1 and NP2 groups but increased in HP1 and HP2 groups (*p* < 0.01) compared with those in the control group. On day 7, P levels were not altered by dietary treatment (*p* > 0.05, **Table 9**). On days 21 and 42, P levels were lower in the NP1 and NP2 groups than in the control group (*p* < 0.01). Within the phytase treatment, an interaction effect was observed between phytase source and level (*p* < 0.01). NP1 had the lowest P level, whereas HP1 and HP2 had the highest P levels. The bone-breaking strength of the tibia was significantly influenced by diet (*p* < 0.01). Compared with the control group, the breaking strength was decreased by all phytase treatments on day 7, increased by HP2 on day 21, and decreased by NP1 on day 42 (**Table 9**). On day 21, the bone-breaking strength was higher in the P2 and HP groups than in the P1 and NP groups (*p* < 0.01). On day 42, the interaction between phytase source and level had a significant influence; NP1 had the lowest breaking strength, whereas HP1 and HP2 had the highest breaking strength (*p* < 0.05).

### 3.5 Femur bone measurements

Dietary treatment had a significant influence on femur length on day 21 (*p* < 0.01), with the HP2 broilers showing a longer length than the control broilers (**Table 10**). On day 21, the P2 and HP treatments significantly increased bone length compared with the P1 and NP treatments (*p* < 0.05). On day 42, the HP treatments significantly increased femur width compared with the NP treatments (*p* < 0.05). Phytase source and level had no obvious influence on femur FW or FFDW at any of the three time points (*p* > 0.05; **Table 10**). Phytase treatment did not alter the BMC and BMD of the femur (*p* > 0.05) on day 7 (**Table 11**). Both phytase source and level influenced BMC or BMD on day 21, and BMC and BMD levels were higher (*p* < 0.01) in the P1 and HP groups than in the P2 and NP groups. However, on day 42, phytase levels significantly changed the BMC and BMD levels (*p* < 0.01), and the HP treatments had higher levels than the NP treatments. Compared with the control group, NP1 and NP2 decreased BMC and BMD levels (*p* < 0.01).

**Table 10:** Effects of phytase sources and levels on femur length, width, fresh weight, fat-free dry weight of broilers fed a diet with non-inorganic phosphorus supplementation.

	Day of age	Dietary treatment^1^							Phytase effect^2^						
		Control	NP1	HP1	NP2	HP2	SEM	*p* value	P1	P2	NP	HP	*p v*alue		
													Source	Level	Source × level
Length (mm)	D 7	30.9	30.5	30.5	30.6	30.4	0.15	0.802	30.5	30.5	30.5	30.4	0.989	0.719	0.748
	D 21	45.8^b^	45.7^b^	46.3^b^	46.5^b^	48.2^a^	0.26	0.007	46.0^y^	47.4^x^	46.1^n^	47.3^m^	0.008	0.029	0.260
	D 42	73.4^a^	69.8^b^	74.0^a^	73.7^a^	73.9^a^	0.58	0.100	71.9	73.8	71.7	74.0	0.155	0.099	0.136
Width (mm)	D 7	2.54	2.46	2.49	2.58	2.35	0.05	0.667	2.48	2.46	2.52	2.42	0.928	0.425	0.297
	D 21	4.18^b^	4.41^ab^	4.29^ab^	4.41^ab^	4.55^a^	0.05	0.242	4.35	4.48	4.41	4.42	0.299	0.923	0.295
	D 42	8.33^ab^	7.73^b^	8.41^ab^	7.89^bc^	8.68^a^	0.10	0.006	8.07	8.28	7.81^n^	8.54^m^	0.269	<0.001	0.781
Fresh weight (g)	D 7	0.82	0.82	0.82	0.80	0.90	0.02	0.570	0.82	0.85	0.81	0.86	0.469	0.246	0.264
	D 21	0.58^ab^	0.62^a^	0.55^b^	0.58^ab^	0.64^a^	0.01	0.037	0.59	0.58	0.60	0.57	0.924	0.300	0.348
	D 42	0.60^ab^	0.64^a^	0.58^b^	0.62^ab^	0.62^ab^	0.01	0.322	0.61	0.60	0.63	0.58	0.686	0.052	0.981
Fat-free dry weight (g)	D 7	0.23	0.21	0.22	0.22	0.23	0.00	0.338	0.23	0.22	0.22	0.23	0.203	0.135	0.957
	D 21	0.18	0.18	0.18	0.18	0.19	0.00	0.940	0.18	0.18	0.18	0.18	0.636	0.647	0.579
	D 42	0.21	0.22	0.20	0.22	0.23	0.00	0.250	0.21	0.22	0.22	0.21	0.185	0.505	0.105

NP1, 1,000FTU/kg phytase 1; HP1, 10,000FTU/kg phytase 1; NP2, 1,000FTU/kg phytase 2; and HP2, 10,000FTU/kg phytase 2.

1 Effect of dietary treatments was evaluated with one-way ANOVA (n=8). ^2^Effects of phytase sources and supplemental levels and their interaction were evaluated with two-way ANOVA. ^a,b^Means without a common superscript in the same row differ significantly (*p* < 0.05). ^m,n^Means with different superscripts between phytase sources differ significantly (*p* < 0.05). ^x,y^Means with different superscripts between phytase levels differ significantly (*p* < 0.05).

**Table 11:** Effects of phytase sources and levels on femur mineral content of broilers fed a diet with non-inorganic phosphorus supplementation.

	Day of age	Dietary treatment^1^							Phytase effect^2^						
		Control	NP1	HP1	NP2	HP2	SEM	*p* value	P1	P2	NP	HP	*p v*alue		
													Source	Level	Source × level
BMC (g)	D 7	0.03	0.02	0.02	0.02	0.02	0.00	0.136	0.02	0.02	0.02	0.02	0.368	0.544	0.798
	D 21	0.20^a^	0.14^b^	0.21^a^	0.08^c^	0.15^b^	0.01	<0.001	0.18^x^	0.12^y^	0.11^n^	0.18^m^	<0.001	<0.001	0.607
	D 42	1.07^a^	0.61^b^	0.93^a^	0.68^b^	0.95^a^	0.03	<0.001	0.77	0.82	0.65^n^	0.94^m^	0.315	<0.001	0.519
BMD (g/cm^2^)	D 7	0.02	0.02	0.02	0.02	0.02	0.00	0.591	0.02	0.02	0.02	0.02	0.977	0.126	0.489
	D 21	0.06^a^	0.04^b^	0.06^a^	0.02^c^	0.04^b^	0.00	<0.001	0.05^x^	0.03^y^	0.03^n^	0.05^m^	<0.001	<0.001	0.945
	D 42	0.11^a^	0.07^b^	0.09^a^	0.07^b^	0.09^a^	0.00	<0.001	0.08	0.08	0.07^n^	0.09^m^	0.733	<0.001	0.432
Ash (%)^3^	D 7	33.2^a^	27.5^c^	31.2^b^	31.7^ab^	32.5^ab^	0.50	<0.001	29.3^y^	32.1^x^	29.6^n^	31.9^m^	<0.001	<0.001	<0.001
	D 21	38.5^ab^	31.5^c^	38.4^ab^	36.4^b^	39.6^a^	0.23	<0.001	34.7^y^	37.7^x^	33.6^n^	39.0^m^	<0.001	<0.001	<0.001
	D 42	39.4^a^	33.7^c^	39.4^a^	37.0^b^	39.0^a^	0.21	<0.001	36.6^y^	38.0^x^	35.3^n^	39.2^m^	<0.001	<0.001	<0.001
Ca (%)^3^	D 7	7.74^a^	6.10^b^	7.73^a^	6.62^b^	8.41^a^	0.58	0.001	6.91	7.52	6.36^n^	8.07^m^	0.089	<0.001	0.799
	D 21	11.5^b^	9.06^c^	13.4^a^	11.1^b^	12.9^a^	0.32	<0.001	11.2^y^	12.0^x^	10.1^n^	13.1^m^	0.011	<0.001	<0.001
	D 42	15.7^b^	13.6^c^	17.3^a^	14.9^bc^	17.7^a^	0.15	<0.001	15.5	16.3	14.3^n^	17.5^m^	0.177	<0.001	0.410
P (%)^3^	D 7	8.41	7.32	8.74	8.38	8.72	0.36	0.187	8.03	8.55	7.85	8.73	0.250	0.061	0.238
	D 21	10.4^a^	8.72^c^	10.6^a^	9.61^b^	10.6^a^	0.33	<0.001	9.68^y^	10.1^x^	9.17^n^	10.6^m^	<0.001	<0.001	<0.001
	D 42	10.6^a^	8.71^c^	10.6^a^	9.63^b^	10.7^a^	0.13	<0.001	9.77^y^	10.1^x^	9.27^n^	10.6^m^	0.009	<0.001	0.018
Breaking strength (N)	D 7	21.5^a^	16.1^b^	15.1^b^	17.9^b^	14.0^b^	0.68	0.002	15.6	15.9	17.0^m^	14.6^n^	0.789	0.045	0.225
	D 21	68.1^b^	51.4^c^	77.1^ab^	69.7^b^	84.0^a^	2.52	<0.001	64.2^y^	76.9^x^	60.6^n^	80.6^m^	0.005	<0.001	0.172
	D 42	258.9^ab^	178.8^c^	300.0^a^	233.6^bc^	290.2^a^	10.5	<0.001	239.4	262.3	206.2^n^	295.4^m^	0.209	<0.001	0.083

NP1, 1,000FTU/kg phytase 1; HP1, 10,000FTU/kg phytase 1; NP2, 1,000FTU/kg phytase 2; and HP2, 10,000FTU/kg phytase 2. BMC, bone mineral content; BMD, bone mineral density. ^1^Effect of dietary treatments was evaluated with one-way ANOVA (n=8). ^2^Effects of phytase sources and supplemental levels and their interaction were evaluated with two-way ANOVA. ^3^Dry matter basis. ^a,b^Means without a common superscript in the same row differ significantly (*p* < 0.05); ^m,n^Means with different superscripts between phytase sources differ significantly (*p* < 0.05). ^x,y^Means with different superscripts between phytase levels differ significantly (*p* < 0.05).

Phytase source and level and their interaction had a significant influence on bone ash content (*p* < 0.01, **Table 11**). The NP1 treatment had the lowest ash content, whereas the HP1 and HP2 treatments had the highest. Compared with the control group, NP1 and HP1 on day 7, NP1 on day 21, and NP1 and NP2 on day 42 had lower ash content (*p* < 0.01). Femoral Ca was significantly influenced by dietary treatment (*p* < 0.01). Compared with the control group, HP1 and HP2 treatments had higher Ca level on day 21 and 42, whereas NP1 and NP2 had lower Ca level at the three time points. On day 7, femoral Ca level was significantly decreased by the HP treatment (*p* < 0.001). On day 21, femoral Ca was influenced by phytase source and level and their interaction (*p* < 0.01). HP1 and HP2 had the highest Ca contents, whereas NP1 had the lowest. The Ca content in the HP broilers was higher than that in the NP broilers (*p* < 0.01). The femoral P content was not influenced by dietary treatment on day 7 (*p* > 0.05, **Table 11**). On days 21 and 42, phytase source and level and their interaction had a significant influence on P content, which was lower in the NP1 and NP2 treatments than in the control group, HP1, and HP2 treatments (*p* < 0.05; **Table 11**). Compared with the control group, the breaking strength of the femur decreased on day 7 in all phytase treatments (*p* < 0.01; **Table 11**). The HP treatment decreased the bone-breaking strength compared with the NP treatment (*p* < 0.05). On day 21, the phytase source and level had a significant influence, and the P2 and HP treatments increased the femur bone-breaking strength (*p* < 0.01). By contrast, breaking strength on day 42 was affected only by phytase supplementation level (*p* < 0.01), with the HP groups showing higher values than the NP groups (**Table 11**).

### 3.6 Expression of genes related to Ca and P metabolism

**Figure 1:**
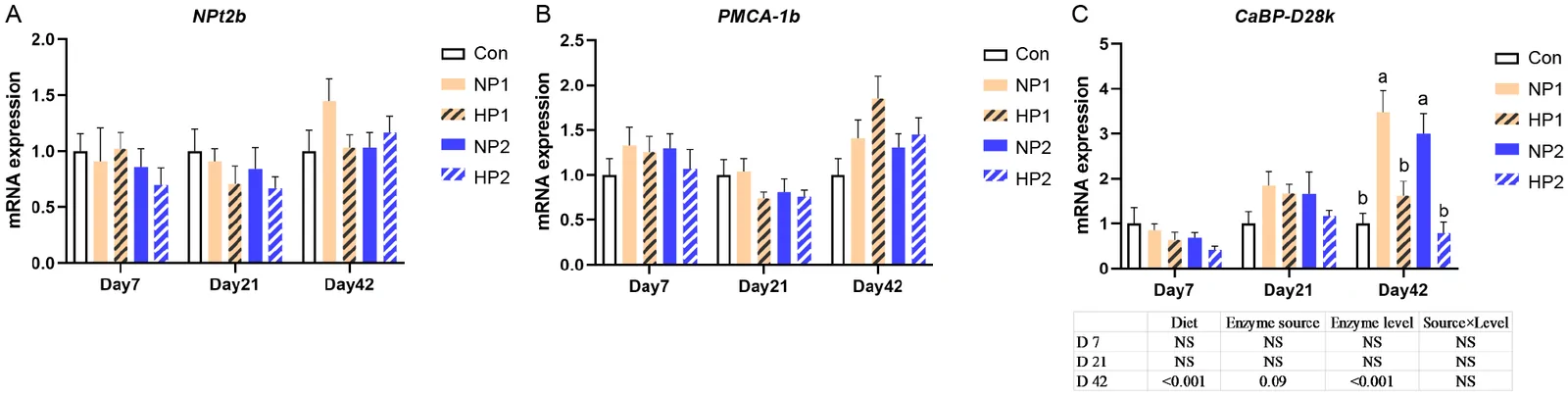
Effects of phytase sources and supplemental levels on the expression levels of *NPt2b* **(A)**, *PMCA-1b* **(B)**, and *CaBP-D28k* **(C)** in the duodenum of broilers fed a diet with non-inorganic phosphate supplementation. Con, control; NP1, 1,000 FTU/kg phytase 1; HP1, 10,000 FTU/kg phytase 1; NP2, 1,000 FTU/kg phytase 2; and HP2, 10,000 FTU/kg phytase 2. ^a,b^Means with different letters differ significantly (*p* < 0.05, n = 8).

In the duodenum, dietary treatment did not alter the expression levels of *NPt2b* and *PMCA-1b* (*p* > 0.05; **Figures 1A, B**). By contrast, *CaBP-D28k* expression did not change after phytase treatment on days 7 and 21 (*p* > 0.05; **Figure 1C**). However, on day 42, the *CaBP-D28k* mRNA level was significantly increased by the NP1 and NP2 treatments compared with that by the HP1 and HP2 treatments (*p <* 0.01). No interaction of phytase source and level was detected (*p* > 0.05).

**Figure 2:**
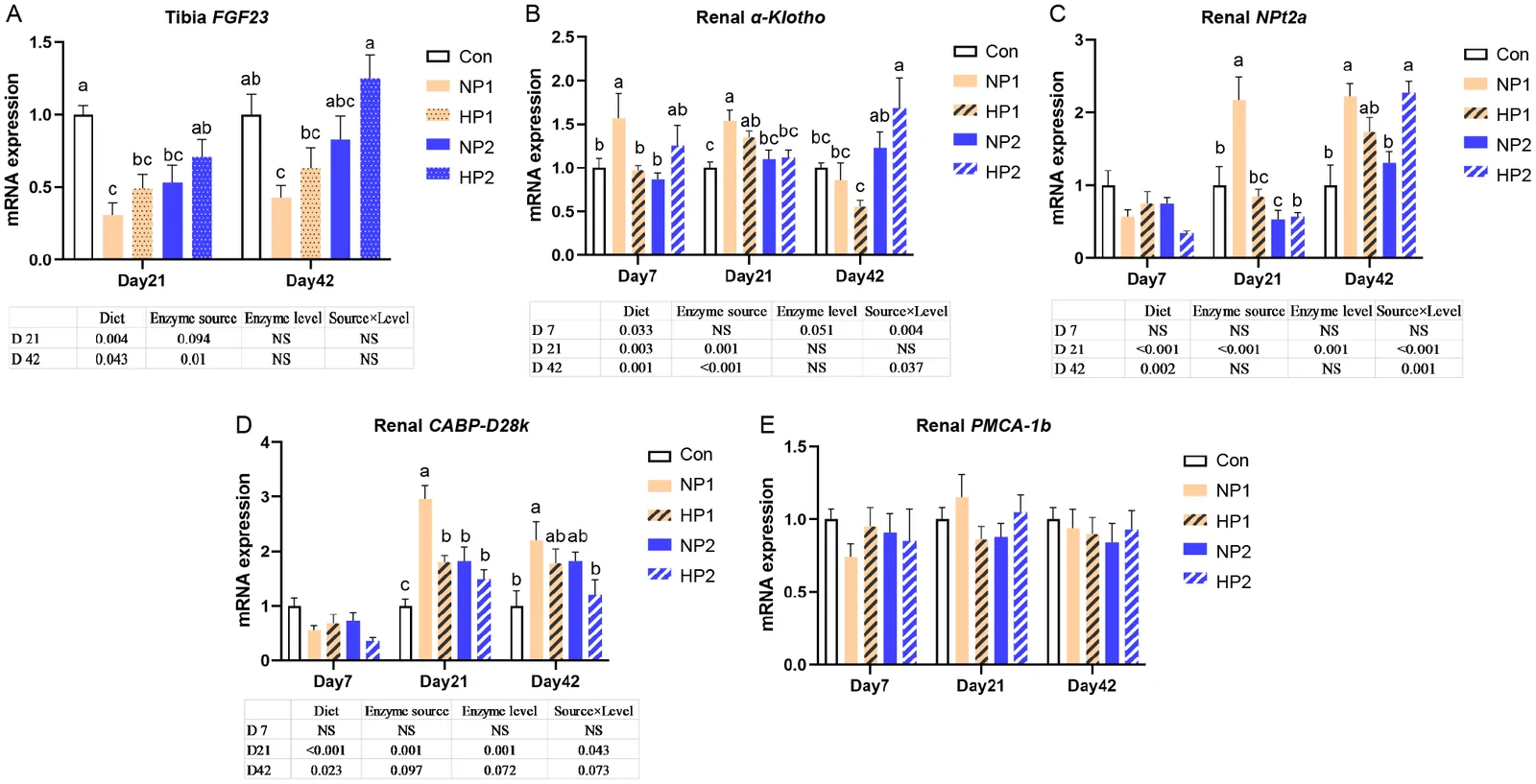
Effects of phytase sources and supplemental levels on the expression levels of *FGF23* in the tibia **(A)** and *α-Klotho* **(B)**, *NPt2a* **(C)**, *CaBP-D28k* **(D)**, and *PMCA-1b* **(E)** in the renal tissues of broilers fed a diet with non-inorganic phosphate supplementation. Con, control; NP1, 1,000 FTU/kg phytase 1; HP1, 10,000 FTU/kg phytase 1; NP2, 1,000 FTU/kg phytase 2; and HP2, 10,000 FTU/kg phytase 2. ^a,b,c^Means with different letter differ significantly (*p* < 0.05, n = 8).

Tibial expression of *FGF23* was significantly influenced by dietary treatment on days 21 (*p* < 0.01) and 42 (*p* < 0.05). Compared with the control group, tibial *FGF23* mRNA expression was significantly decreased in NP1, HP1, and NP2 on day 21 and in NP1 on day 42 (**Figure 2A**). Phytase source significantly affected *FGF23* expression on day 42, with higher expression in P2 than in P1 (*p* = 0.01), whereas phytase level and the source × level interaction had no significant effects (*p* > 0.05). In the kidney, compared with the control group, the expression of *α-Klotho* was significantly increased by NP1 on day 7 (*p* < 0.05), NP1 and HP1 on day 21 (*p* < 0.01), and HP2 on day 42 (*p* < 0.01; **Figure 2B**). Phytase source had an influence (*p* < 0.01) on the expression of *α-Klotho*, with higher expression levels in P1 than in P2 on day 21, whereas the opposite was true on day 42. There was an interaction of phytase source and level on the expression of *α-Klotho* (*p* < 0.05), which was higher in NP1 on day 7 and in HP2 on day 42.

In the kidney, *NPt2a* and *CaBP-D28k* mRNA levels were not altered by treatment on day 7 (*p* > 0.05; **Figures 2C, D**). Compared with the control group, renal *NPt2a* mRNA expression was increased in NP1 and decreased in NP2 on day 21, whereas it was increased in NP1 and HP2 on day 42 (**Figure 2C**). By contrast, compared with control, *CaBP-D28k* expression was increased by all phytase treatments on day 21 (*p* < 0.001) and by NP1 on day 42 (*p* < 0.05). Phytase source, level and their interaction all had a significant influence on the expression of *CaBP-D28k* on day 21 (*p* < 0.05), with a higher level in NP1 than in the other groups. By contrast, phytase treatment had no significant effect on day 42 (*p* > 0.05). *PMCA-1b* expression remained unchanged across all treatments (*p* > 0.05; **Figure 2E**).

## 4 Discussion

Phytase improves P availability by hydrolyzing phytate and releasing phytate-bound P, thereby enhancing P utilization in broilers. The present study showed that phytase supplementation, particularly at 10,000 FTU/kg, largely offset the effects of removing supplemental inorganic P from broiler diets. High-dose phytase reduced fecal P excretion while maintaining growth performance and bone characteristics comparable to those of birds fed the nutritionally adequate control diet. These findings highlight the potential of high-dose phytase to improve phytate P utilization and support broiler production under diets without supplemental inorganic P.

Growth performance responded clearly to phytase supplementation level. Broilers receiving 10,000 FTU/kg phytase maintained FI, BW gain, and FCR at levels comparable with the control. In contrast, 1,000 FTU/kg reduced FI and BW gain, while the FCR response varied with phytase source. Similar dose-dependent improvements have been reported in broilers fed P-reduced or inorganic-P-free diets (Kriseldi et al., 2021a; Marchal et al., 2021; Jlali et al., 2024; Sobotik et al., 2024). The greater efficacy of high-dose phytase may result from more extensive phytate degradation and improved nutrient availability. Increasing the phytase dose promotes phytate degradation and myo-inositol release (Kriseldi et al., 2021b). It can also improve amino acid digestibility and P utilization (Dersjant-Li et al., 2022). These findings support the greater efficacy of high-dose phytase in maintaining growth performance when supplemental inorganic P is omitted.

The responses of P and Ca utilization to phytase dose varied with the measured index and growth stage. During the starter phase, P retention was greater in HP than in NP broilers, whereas apparent P retention did not differ between phytase levels. The greater absolute P retention in HP birds may partly reflect their higher feed intake, as P intake also tended to be greater with HP supplementation. In the finisher phase, neither P retention nor apparent P retention differed between NP and HP treatments. Nevertheless, serum P concentrations on days 21 and 42 and tibial and femoral P contents were generally higher in HP than in NP broilers, indicating a more favorable P status with the higher phytase dose. These findings are consistent with previous studies showing that phytase supplementation improves P digestibility and bone mineralization in broilers fed P-deficient diets (Jlali et al., 2024; Sung et al., 2024), as well as with evidence that high-dose phytase increases intestinal P disappearance (Ajuwon et al., 2020). Ca retention was unaffected by dietary treatment during the starter phase. During the finisher phase, Ca retention was lower only in NP1 than in the control group, while factorial analysis showed greater Ca retention in HP than in NP and in P2 than in P1 broilers, indicating that the effects of phytase on Ca utilization at the later growth stage were related to both supplementation level and phytase source.

Serum Ca, P, and PTH responses were influenced by phytase supplementation level, age, and phytase source. In particular, serum P and PTH concentrations were higher in HP than in NP broilers on days 21 and 42. Previous studies have also shown that phytase supplementation can modulate circulating Ca, P, and PTH concentrations in broilers fed P-deficient diets (Nari and Ghasemi, 2020), and beneficial effects of high-dose phytase under Ca- and P-deficient conditions have similarly been reported (Bassi et al., 2025). Serum CT showed a transient response to phytase supplementation, with lower concentrations in HP broilers than in the control group on day 7, whereas no clear treatment response was evident at later ages, consistent with the generally limited responsiveness of circulating CT to phytase supplementation reported in P-deficient broilers (Nari and Ghasemi, 2020).

Mineral deposition and bone development were also influenced by phytase supplementation. Reductions in bone ash, BMC, and BMD were observed mainly in the NP treatments, whereas HP generally maintained or improved these indices, particularly on day 42. Similar dose-related improvements in bone mineralization and breaking strength have been reported in broilers fed P-reduced diets (Babatunde et al., 2021; Sobotik et al., 2024; Sung et al., 2024). Bone-breaking strength was reduced across phytase treatments on day 7, whereas HP generally improved bone strength at later ages. This early reduction may indicate that phytase-released P was insufficient to fully support the rapid skeletal mineralization required during the first week of life. Differences between the femur and tibia may also reflect their distinct developmental patterns (Applegate and Lilburn, 2002). These findings suggest that diets without supplemental inorganic P may not fully meet the early P requirement for bone development, even with phytase supplementation. Differences in bone ash and breaking strength between P1 and P2 further suggest source-specific variation in phytase efficacy, consistent with previous reports of differences among phytase products in their effects on bone mineralization and mineral utilization (Bowen et al., 2022; Zhang et al., 2022). Overall, high-dose phytase was more effective in supporting bone development and mineralization after the first week of age.

Serum PINP and PICP are commonly used as indices of type I collagen formation and bone matrix synthesis, whereas TRAP-5b reflects osteoclast-associated bone resorption (Price et al., 2017; Seo et al., 2017; Ge et al., 2024). Studies in broilers have further shown that dietary P restriction alters skeletal and systemic mineral responses, whereas phytase supplementation in P-reduced or inorganic-P-free diets improves P utilization and can preserve or enhance bone mineralization and bone quality (Marchal et al., 2021; Dersjant-Li et al., 2022; Cozannet et al., 2023; Omotoso et al., 2023; Shi et al., 2024). In the present study, osteocalcin decreased only on day 21 in NP2, whereas PINP decreased on day 42 with all phytase treatments. BALP showed no consistent response to dietary treatment, although it was lower in HP than in NP broilers on day 21. PICP and TRAP-5b showed treatment- and age-dependent responses rather than consistent changes across phytase treatments. Collectively, these findings indicate that phytase supplementation in diets without inorganic P did not produce a sustained shift in circulating markers toward either bone formation or bone resorption.

At the molecular level, duodenal *NPt2b* mRNA expression remained unchanged, whereas renal *NPt2a* expression showed treatment-specific responses, consistent with reports that renal *NPt2a* is more responsive than duodenal *NPt2b* to dietary NPP and phytase (Ma et al., 2025). Tibial *FGF23* mRNA expression was generally reduced by diets without supplemental inorganic P, particularly at earlier ages, while renal *NPt2a* expression increased in some treatments. This inverse response may reflect an adaptation favoring renal P conservation, although it was not consistent across treatments. FGF23 regulates P homeostasis through FGF receptors with α-Klotho as a co-receptor, and both FGF23 and α-Klotho are expressed in tissues involved in P homeostasis in chickens (Magnuson et al., 2024; Meier et al., 2025). The variable *α-Klotho* response further suggests that these transcriptional changes depend on age and phytase treatment. In addition, the unchanged or increased expression of *CaBP-D28k* and *PMCA-1b*, together with largely maintained Ca retention, suggests that intestinal Ca transport capacity was not markedly impaired. *CaBP-D28k* and *PMCA-1b* are involved in intestinal Ca transport. In broilers, their mRNA expression is influenced by dietary mineral and vitamin D status and may also respond to interactions between dietary NPP and phytase (Han et al., 2024; Ma et al., 2025).

Future studies incorporating gradient phytase levels and a negative control group without phytase addition may help define the appropriate phytase level required in the diet without inorganic P supplementation. Moreover, a 23-h lighting and 1-h darkness regime was used. In medium-growing broiler chickens, the BW and feed intake of chickens responded to light period (12- to 24-h lighting) in a linear fashion, while shank length and abdominal adipose tissue weight showed a quadratic response to light period (Yang et al., 2015). Hence, the possible influence of lighting regime should be investigated further.

## 5 Conclusions

In conclusion, supplementation with high-dose phytase (10,000 FTU/kg) maintained broiler growth performance and improved P utilization, bone mineral deposition, and skeletal development in diets without supplemental inorganic P. These findings indicate that high-dose phytase has the potential to substantially replace inorganic P supplementation, although its efficacy may vary among phytase sources.

## Data Availability

The original contributions presented in the study are included in the article/supplementary material. Further inquiries can be directed to the corresponding authors.
